# 18q22.1-qter deletion and 4p16.3 microduplication in a boy with speech delay and mental retardation: case report and review of the literature

**DOI:** 10.1186/s13039-018-0404-2

**Published:** 2018-10-19

**Authors:** Chunjing Wang, Huanhuan Ren, Huaifu Dong, Meng Liang, Qi Wu, Yaping Liao

**Affiliations:** 1 Department of Life Sciences, Bengbu Medical College, 2600 Donghai Avenue, Bengbu, Anhui 233030 People’s Republic of China; 2 Department of Paediatrics, The First Affiliated Hospital of Bengbu Medical College, 2600 Donghai Avenue, Bengbu, Anhui China

**Keywords:** 18q22.1 deletion, 4p16.3 duplication, Speech delay, Mental retardation, Facial dysmorphisms

## Abstract

**Background:**

Deletions involving the long arm of chromosome 18 have been associated with a highly variable phenotypic spectrum that is related to the extent of the deleted region. Duplications in chromosomal region 4p16.3 have also been shown to cause 4p16.3 microduplication syndrome. Most reported patients of trisomy 4p16.3 have more duplications, including the Wolf-Hirschhorn critical region (WHSCR). Here, we present a patient with speech delay and mental retardation caused by a deletion of 18q (18q22.1-qter) and terminal microduplication of 4p (4p16.3-pter) distal to WHSCR.

**Case presentation:**

The patient was a 23-month-old boy with moderate growth retardation, severe speech delay, mental retardation, and dysmorphic features. Single nucleotide polymorphism (SNP) array analysis confirmed an 11.2-Mb terminal deletion at 18q22.1 and revealed a 1.8-Mb terminal duplication of 4p16.3. Our patient showed clinical overlap with these two syndromes, although his overall features were milder than what had been previously described. Some dosage-sensitive genes on the 18q terminal deleted region and 4p16.3 duplicated region of the present case may have contributed to his phenotype.

**Conclusions:**

This is the first report of a patient with combined terminal deletion of 18q22.1 and duplication of 4p16.3. In this report, we provide clinical and molecular evidence supporting that the microduplication in 4p16.3, distal to WHSCR, is pathogenic. The coexistence of two chromosome aberrations complicates the clinical picture and creates a chimeric phenotype. This report provides further information on the genotype-phenotype correlation of 18q terminal deletion and 4p microduplication.

## Background

Deletions of the long arm of chromosome 18 (18q deletion syndrome, OMIM #601808) are common abnormalities involving chromosome 18 [1]. Individuals with a terminal 18q deletion display variable phenotypes, including short stature, microcephaly, characteristic dysmorphic facial feature, cleft lip/palate, delayed myelination, foot deformities, hypotonia, congenital aural atresia (CAA), mental retardation (MR), and genitourinary malformations [2–5]. Some dosage-sensitive genes and critical regions on 18q that contribute to the clinical features have been identified, thereby providing a foundation for establishing the genotype-phenotype correlation for 18q deletions [1, 6].

Terminal deletions of chromosome 4p cause Wolf-Hirschhorn syndrome (WHS, OMIM #194190). Duplications involving 4p16.3 have also been reported in several individuals, giving rise to a proposed 4p16.3 microduplication syndrome [7, 8]. Larger imbalanced rearrangements on chromosome 4p in the form of deletions and duplications involving the Wolf–Hirschhorn critical region (WHSCR) have defined clinical features, such as developmental delay, delayed psychomotor development, intellectual disability, and craniofacial and skeletal malformations [9–12]. However, the significance and clinical presentation of patients with microduplication distal to WHSCR are not well understood. To the best of our knowledge, only two patients have been reported to date [8, 13].

Here, we report another case of a 23-month-old boy with a 1.8-Mb duplication at 4p16.3 distal to the WHSCR combined with an 11.2-Mb terminal deletion at the 18q22.1, who presents with similar and different symptoms previously seen in trisomy 4p and 18q deletion syndrome. This case provides further information regarding the clinical features and genotype-phenotype correlation of 18q deletion and 4p16.3 microduplication.

## Case presentation

The patient was a 23-month-old boy who was referred for cytogenetic studies because of speech delay and mental retardation. He was born at 38 weeks gestation following an unremarkable pregnancy by Caesarean section. His birth weight was 3.40 kg (<50th centile), and birth length was 52 cm (>75th centile). At birth, he had an umbilical hernia, which healed at 3 months of age.

The patient could sit at 8 months, and took his first steps at 18 months. At 23 months, his height was 90 cm (<75th centile), and his weight was 13 kg (<75th centile). He cannot speak meaningful words and walked with instability and large strides. Medical examination revealed developmental delay, sensory integration dysfunction, moderate MR, and reduced cognitive ability. Additional physical features included hypotonia, a moon face, midface hypoplasia, deep-set eyes, epicanthal folds, a wide nasal bridge, a flat nose, a protrusible mouth, short neck, and a longer fourth toe of the right foot. No significant defects such as cleft lip/palate, ears, heart, lung or genitourinary system were noted.

## Materials and methods

### Karyotyping

For chromosome analysis, metaphase chromosomes were obtained from peripheral blood lymphocytes after 72 h of incubation and were prepared for GTG banding according to standard protocols.

### Fish

According to standard protocols, the cultured blood lymphocytes of the patient were harvested to obtain metaphase chromosomes. FISH analysis was performed with six different chromosome 18 BAC probes; RP11-7H17, 185,880 bp, 18q23 (chr18:77,115,373-77,301,252); RP11-55 N14, 173,736 bp, 18p11.31 (chr18:2,744,753-2,918,488); RP11-53 N15, 678 bp, 18q22.3 (chr18:71,881,306-71,881,983); RP11-90 L7, 165,866 bp, 18q11.2 (chr18:23,355,089-23,520,954); RP11-79A24, 141,894 bp, 18q22.1 (chr18:66,593,997-66,735,890); and RP11-90E1, 182,823 bp, and 18q12.3 (chr18:41,350,363-41,533,185). Images were captured using a fluorescent microscope (Leica DM5000B), and signals were analyzed using Leica CW 4000 software.

### SNP array analysis

Infinium OmniZhongHua-8 SNP arrays (Illumina, San Diego, CA, USA) were utilized to analyze genome-wide copy number aberrations. DNA amplification, tagging, and hybridization were processed according to manufacturer’s protocols. Illumina’s iScan system was used to scan the beadchips. Image data was analyzed using Illumina’s GenomeStudio.

## Results

**Fig. 1:**
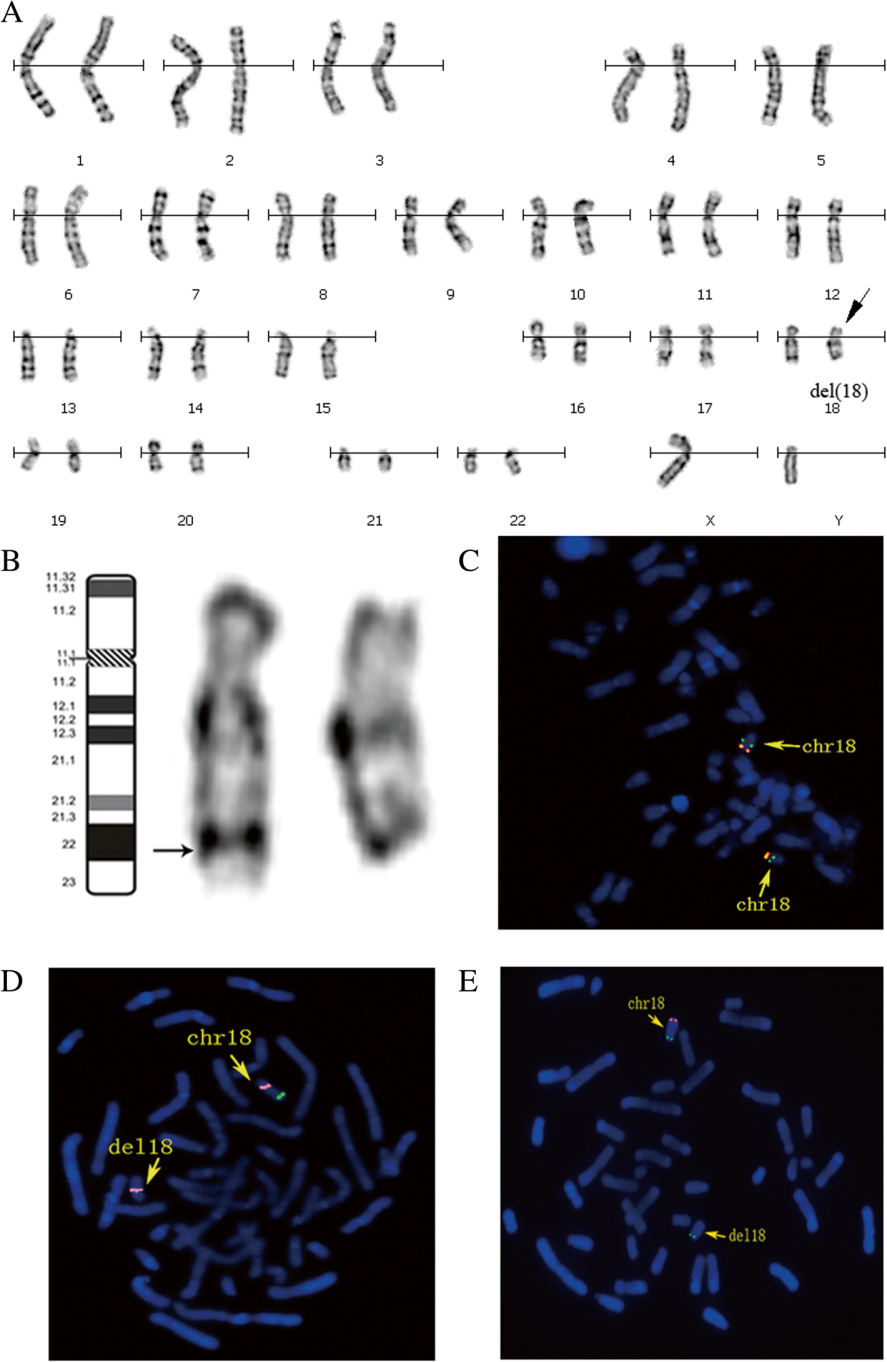
Identification of chromosome 18 terminal deletion. **a** GTG banded karyotype of the case showing an aberration at distal 18q indicated by an arrow. **b** G-banding of chromosome 18 showing the deletion of 18q. **c** BAC-probes RP11-79A24 (orange) (18q22.1) (chr18:66,593,997-66,735,890) and RP11-90E1 (green) (18q12.3) (chr18:41,350,363-41,533,185) showing no deletion at 18q22.1 (chr18:66,593,997-66,735,890). **d** BAC-probes (green) (18q22.3) (chr18:71,881,306-71,881,983) and RP11-90 L7 (orange) (18q11.2) (chr18:23,355,089-23,520,954) revealing the deletion of 18q22.3. **e** BAC-probes RP11-7H17 (orange) (18q23) (chr18:77,115,373-77,301,252) and RP11-55 N14 (green) (18p11.31) (chr18:2,744,753-2,918,488) revealing the deletion of 18q23

**Fig. 2:**
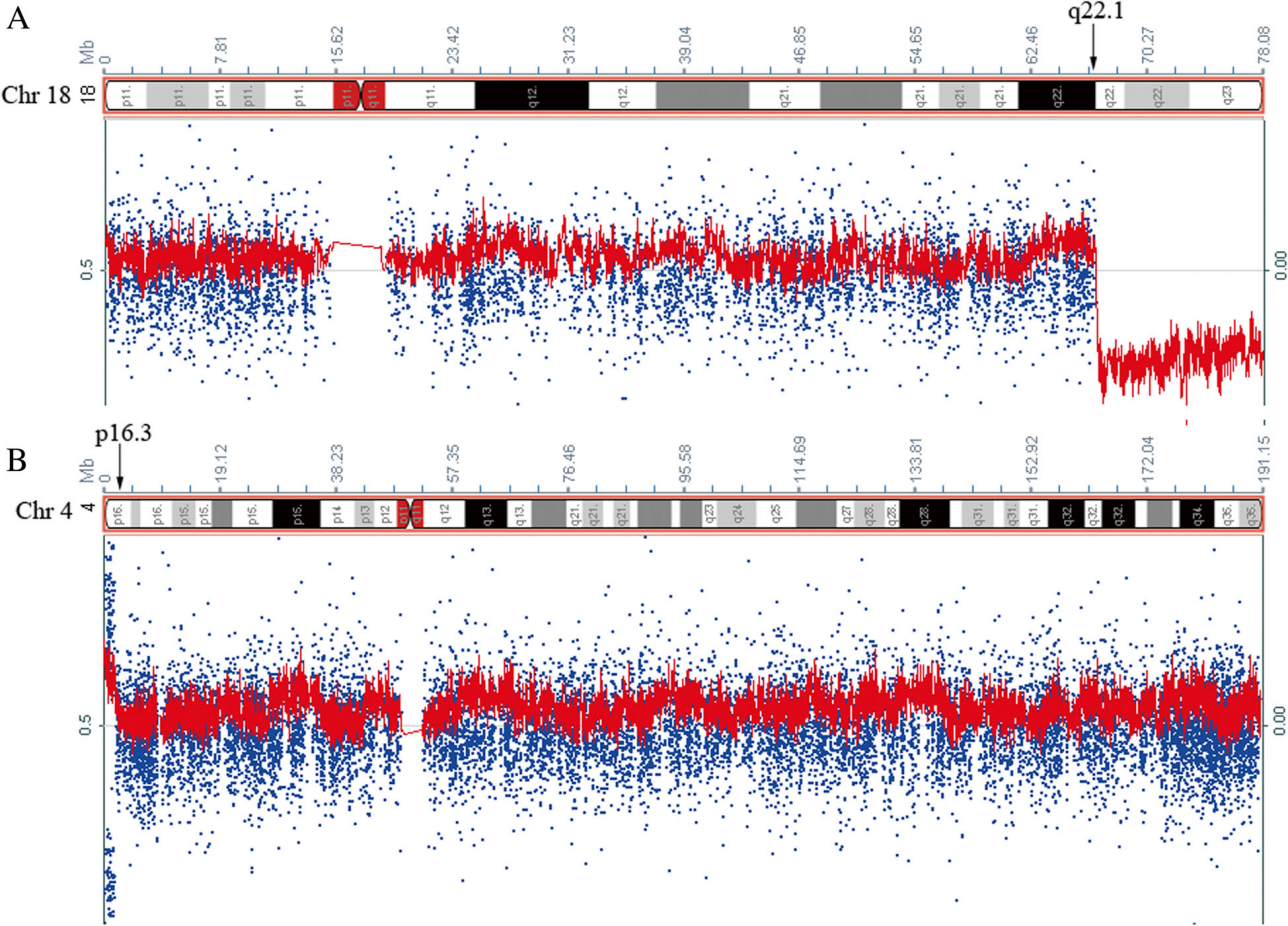
SNP array-based chromosome analysis. **a** Identification of the deletion in chromosome 18 revealed an 11.2-Mb deletion at 18q22.1 (chr18:66,794,478–78,015,180). **b** Revealing a 1.8-Mb duplication at 4p16.3 (chr4:71,566–1,883,647)

The G-banding chromosomal analysis revealed a karyotype of 46, XY, del (18) (q22) (Figs. 1a and b). The karyotypes of his parents are normal. To investigate the deletion region of 18q, fluorescence in situ hybridization (FISH) analysis using chromosome 18 BAC probes was performed. Data showed hybridization signals in the region of 18q22.1 (chr18:66,593,997-66,735,890) (Fig. 1c) and no hybridization signal in the region of 18q23 (chr18:77,115,373-77,301,252) (Fig. 1d) and 18q22.3 (chr18:71,881,306-71,881,983) (Fig. 1e), indicating an 18q22 terminal deletion. To identify the size and position of the chromosomal aberrations, we performed SNP array analysis. The results showed a terminal deletion at 18q22.1 (Fig. 2a) and revealed the presence of a duplication at 4p16.3 (Fig. 2b). The terminal deletion 18q22.1 was approximately 11.2 Mb (66,794,478–78,015,180) in size and the 4p16.3 duplication was approximately 1.8 Mb (71,566–1,883,647) in size (UCSC genome browser, Human Assembly, GRCh37/hg19).

## Discussion

Using conventional karyotyping, FISH, and SNP array analyses, we report a case involving a terminal deletion of 18q22.1 and duplication of 4p16.3. Patients with terminal deletions of the long arm of chromosome 18 or microduplications of the short arm of chromosome 4 display diverse phenotypes. The clinical phenotypes of our patient show similarities and differences from previously reported cases.

The 18q22 deletion syndrome is characterized by mental retardation and development delay, as well as a range of physical anomalies, including microcephaly, short stature, hearing loss, CAA, cleft palate with or without cleft lip (CL/CP), white matter abnormalities of the brain, hand and foot malformations. Several reports have explored the genotype-phenotype correlations of 18q22 deletion syndrome. Recently, Cody et al. [6] viewed more than 350 individuals with 18q22 deletion syndrome and classified 133 of a total of 196 confirmed genes on 18q as dosage-insensitive and 15 (8%) as dosage-sensitive leading to haploinsufficiency, whereas another 10 (5%) have effects that are conditionally haploinsufficient and dependent on another factor, such as genetic or environmental, to cause an abnormal phenotype. Our patient’s deletion region includes 5 of the 15 genes. Those genes are *NETO1* (chr18:70,409,549-70,534,810), *CYB5A* (chr18:71,983,110-72,026,422), *TSHZ1* (chr18:72,997,498-73,000,596), *MBP* (chr18:74,690,789-74,844,774), and *NFATC1* (chr18:77,160,326-77,289,323).

Although the terminal deletion involving 18q was approximately 11.2 Mb in size and included five dosage-sensitive genes, our patient’s clinical features were relatively mild and distinct from those described in 18q- syndrome. Abnormal phenotypes in our case were only associated with *NETO1* and *MBP* genes. In terms of *NETO1* (chr18:70,409,549-70,534,810), patients who are hemizygous for this gene represent executive function difficulties [14]. Haploinsufficiency of this gene has been associated with impaired spatial learning and memory. The *MBP* gene lies within the 18q23 CNS dysmyelination critical region (chr18:72,980,819-75,485,284), and hemizygosity of the *MBP* gene can cause dysmyelination of the brain for people with distal 18q- [14, 15]. Our patient presents MR, walking instability, and large strides, which may be attributed to CNS dysmyelination. However, high-frequency sensorineural hearing loss, which has been associated with the *MBP* gene, was not observed in our patient. In addition, defects in the *TSHZ1* gene would cause cleft soft palate and CAA [16], but our patient does not exhibit both symptoms, although the deletion region of this case included this gene. The other case with a larger deletion of 18q21.3-qter that we previously reported also did not present sensorineural hearing loss, CAA, and CL/CP [17]. Some cases presenting with a mild phenotype suggest that features associated with this deletion is highly heteregeneous. It seems that hemizygosity of some dosage-sensitive genes, such as *TSHZ1* and *MBP,* may not lead to an abnormal phenotype. We infer that variable phenotypes may be attributed to potential gene-gene interactions and gene-environmental interactions.

Interestingly, our patient presents severe speech delay, although the deleted region in 18q does not include the dosage-sensitive gene *SETBP1* (chr18:42,260,863-42,648,475), which is associated with severe delay in expressive speech with intact receptive language [6, 18, 19]. It may be due to a 1.8-Mb duplication involving 4p16.3 that was also observed in this case. Two critical regions within 4p16.3-WHSCR1 and WHSCR2 for WHS were identified [20, 21]. Most patients with trisomy 4p16.3 have more duplications, which include WHSCR1 and WHSCR2. In contrast, smaller duplications in 4p16.3 distal to WHSCR1 and WHSCR2 are rare. To the best of our knowledge, only two patients have been reported to have this defect [8, 13]. In addition, the duplication involving chromosome 4p16.3 in our patient does not involve WHSCR2 and the coding region of WHSCR1. His clinical features also overlap with the two previously reported cases, including developmental delay, dysmorphic facial features, and speech and cognitive delay. Clinical features of the three patients are discussed in detail and summarized in Table 1.

**Table 1:** Clinical features of the duplication 4p16.3 cases

Clinical features	Present case	Cyr et al. [13]	Palumbo et al. [8]
Duplication positions	71,566–1,883,647	1,326,373-1,832,617	1,405,662-1,798,461
Other chromosome anomalies	Terminal deletion of 18q22.1	–	–
Gestational age (weeks)	39	38–4/7	Unknown
Sex and age at diagnosis	M, 23 months	M, 9 months	M, 13 years
Weight	< 50th centile	30th centile	> 97th centile
Height	> 75th centile	30th centile	90-97th centile
Head circumference	> 75th centile	> 95th centile	25-50th centile
Neurologic			
Speech delay	Yes (severe)	Yes	Yes
Developmental delay	Yes	Yes	Yes
seizure	No	Yes	No
Sensory Integration	Dysfunction (squinting while running)	Unknown	Dysfunction (ADHD)
Short stature/failure to thrive	No	Yes	No
MRI	Unknown	Dilatation of the lateral ventricles	Normal
Craniofacial			
Macro/microcephaly	No	Yes (Macrocephaly)	No
Frontal bossing	Yes	Yes	Yes
Hypertelorism	Yes	Yes	Yes
Epicanthal folds	Yes	Yes	Yes
palpebral fissures	Normal	Narrow and long	Downslanted
Eyes	Normal	Iris heterochromia; hyperopia	Hyperopia
Ears	Low-set and dysmorphic	Low-set and posteriorly rotated	Normal
Nose	Broad nasal root and short nasal bridge	Broad nasal root and short nasal bridge	Normal
Palate	Normal	Normal	High arched
Retrognathia/micrognathia	No	No	Yes
Neck	Short	Short	Short
Musculoskeletal			
Hypotonia/ Hypertonia	Yes (Hypotonia)	No	No
Balance difficulty	Yes	No	Unknown
Upper/Lower extremity	Longer fourth toe of the right foot	Bridged palmar crease, syndactyly,	Bilateral flatfoot
Others	Umbilical hernia	Prominent fetal pads; slightly more hair in the lumbosacral region	Scoliosis, dental abnormalities, gynecomastia

*ADHD* Attention deficit hyperactivity disorder

Some dosage-sensitive genes, such as *LETM1*, *WHSC1*, and *WHSC2,* can be responsible for the duplication 4p phenotype [22]. However, the phenotypic spectrum of patients without the involvement of the WHS critical region is not well understood [13]. The 1.8-Mb microduplication in the present case includes at least 34 RefSeq genes, and some of the genes in this region may have a role in the 4p trisomy phenotype, which include *TACC3, FAM53A, FGFR3, LETM1, SLBP*, and *CRIPAK*. TACC3 (transforming acidic coiled-coil containing protein 3) is involved in microtubule dynamic regulation during cell division. Microtubule dynamics are essential to mitotic spindle assembly for appropriate neurogenesis in the cerebral cortex [23, 24]. Peset and Vernos [25] showed that the overexpression of *TACC3* cause defects in chromosome alignment and eventually mitotic arrest. Piekorz et al. [26] reported that TACC3 is a critical component of the centrosome/mitotic spindle apparatus, and its absence triggers p53-mediated apoptosis. Based on this evidence, we hypothesize that the overexpression of *TACC3* influences early embryonic neural development by interfering with neuronal apoptosis and/or cell cycle. Another gene, *FAM53A*, is also known as a candidate gene for neurodevelopmental features because it is highly expressed in the early embryonic central nervous system, suggesting that it plays critical roles in neuronal development [8, 13]. In summary, neurodevelopmental delay, which we have described in this case study and in other patients, [13, 22] may be caused by an increased dosage of the *TACC3* and *FAM53A* genes.

Another key gene of the 4p16.3 duplication may be *LETM1.* It encodes a leucine zipper EF hand-containing transmembrane protein 1 that is involved in mitochondrial morphology, protein transport, and mitochondrial K^+^/H^+^ exchange [27, 28]. It has been suggested that the overexpression of *LETM1* can cause seizures [8, 13, 22, 29], but our patient did not exhibit this symptom. No duplication carriers of a three-generation family described by Schönewolf-Greulich’s group [12] experienced seizures either. Data from these patients with 4p16 duplications suggest variable penetrance of epilepsy [8, 13, 22, 29]. It is not clear how duplication of the *FGFR3*, *CRIPAK*, and *SLBP* genes could affect the phenotype expressed in our patient. Evaluation of additional patients with well-characterized 4p16.3 duplication and/or point mutations in this region will be useful to illuminate the role of individual genes in these clinical features.

In addition, severe speech delay is the outstanding clinical symptom of our patient, which has also been observed in the other two individuals. As of writing, the patient is nearly 4 years old. We followed the patient and found that he could not speak complete sentences. Hannes et al. [22] suggested that speech development may be impaired by the aberration involving the WHSCR, whereas the duplicated region of the three individuals does not include WHSCR. Although these evidences suggest there may be a candidate region or genes, such as *TACC3*, *FAM53A*, and *LETM1,* shared by all three patients associated with speech delay on 4p16.3 distal to WHSCR, additional experimental and clinical data are needed to support our hypothesis.

## Conclusions

We describe a 23-month-old male with a combined terminal deletion of 18q22.1 and duplication of 4p16.3. Compared to other individuals with 18q22.1 deletions, our patient presents a relatively mild phenotype. The coexistence of two chromosomal rearrangements complicates the clinical symptoms and creates a chimeric disorder marked by characteristics of both chromosomal abnormalities. Additionally, our case report provides clinical and molecular evidence supporting the existence of a novel 4p16.3 microduplication syndrome. The *TACC3* and *LETM1* genes apparently play a key role in the etiology of the clinical phenotype of 4p16.3 microduplication.

## Abbreviations

FISH: Fluorescence in situ hybridization
SNP array: Single nucleotide polymorphism array
WHS: Wolf–Hirschhorn syndrome
WHSCR: Wolf-Hirschhorn critical region
